# Artificial intelligence-driven food and nutrition systems: from smart food production to personalized nutrition

**DOI:** 10.3389/fnut.2026.1922164

**Published:** 2026-08-05

**Authors:** Xi Chen, Zimeng Cheng, Wenshuo Wang, Kailin Zhong, Bin Xue

**Affiliations:** 1 Guangdong-Hong Kong-Macao Greater Bay Area College of Intelligent Cold-Chain Industries, Guangzhou College of Technology and Business, Guangzhou, Guangdong, China; 2 College of Food Science, South China Agricultural University, Guangzhou, Guangdong, China

**Keywords:** artificial intelligence, digital health, food manufacturing, food systems, personalized nutrition, smart agriculture, sustainability

## Abstract

Artificial Intelligence (AI) is rapidly emerging as a technology that integrates sectors such as food production, manufacturing, supply-chain management, diet-related information, and personalized nutrition. By integrating machine learning, computer vision, predictive analytics, robotics, and large language models, AI is reshaping how food is produced, processed, evaluated, and consumed. In primary production, it enhances precision agriculture and resource optimization. During processing and distribution, AI-driven automation, quality control, and predictive systems enhance efficiency, safety, and supply-chain resilience. At the consumer interface, adaptive dietary recommendation systems integrate user profiles, food composition databases, clinical biomarkers, omics profiles, and behavioral feedback to generate personalized recommendations. Emerging hybrid AI approaches that combine computational intelligence with established nutritional science are also improving dietary assessment. For example, image-based systems using convolutional neural networks and transformer architectures demonstrate promising capabilities in automated food recognition and intake estimation. Despite these advances, AI-driven food and nutrition systems remain in an early translational phase, with substantial challenges limiting widespread adoption. These include heterogeneous and incomplete datasets, limited real-world validation, insufficient model transparency, regulatory and ethical uncertainties, and unequal accessibility among small-scale producers and vulnerable populations. Addressing these barriers will require interdisciplinary collaboration among food scientists, nutritionists, data scientists, policymakers, and industry stakeholders. This perspective examines the emerging role of AI as a transformative force in nutritional intelligence, smart food manufacturing, sustainable food systems, and precision nutrition. The article also discusses key opportunities and challenges for its responsible integration into future food systems.

## 1 Introduction

Food and nutrition systems are under increasing pressure to produce and distribute nutritious food in ways that are resilient, resource-efficient, equitable, and environmentally sustainable. This challenge is expected to intensify as the global population approaches 10 billion by 2050 (1, 2), increasing demand for safe, nutritious, and affordable food while placing additional strain on natural resources. At the same time, unhealthy diets remain one of the leading preventable risk factors for non-communicable diseases and are responsible for millions of deaths each year. Cereal demand is projected to reach around 3 billion tons, requiring an additional ∼1 billion tons of annual production compared with today. Meat production will also need to rise by over 200 million tons to meet a projected total of 470 million tons, with most of this growth occurring in developing countries (2). Moreover, current dietary habits also place heavy pressure on the environment, highlighting the need for coordinated action across the entire food system (3).

Another major challenge is that population-level dietary guidelines often fail to account for the substantial variation in individual responses to food, which are influenced by genetics, metabolism, health status, lifestyle, culture, food access, and environmental factors (4). To address this complexity, computational nutrition has emerged as an interdisciplinary field that integrates statistical modeling, systems simulation, machine learning, and deep learning to understand and predict diet–health interactions (5). Unlike conventional nutrition data analytics, computational nutrition combines dynamic modeling with large-scale biological and behavioral data to support personalized dietary recommendations, predict individual metabolic responses to foods, and establish individualized dietary reference intakes.

Furthermore, personalized nutrition advice significantly improved Mediterranean Diet scores compared to general dietary advice, with the addition of genotype-based advice producing the largest differences (6). Livingstone and Celis-Morales conducted a study involving 1,607 adults across seven European countries to determine who benefits most from internet-based personalized nutrition interventions. After 6 months, greater improvements in diet quality and health-related outcomes were observed primarily among older adults, women, those with poor baseline diets, and individuals who were more motivated and confident about adopting healthy eating habits (7). These findings highlight the need for more adaptive, individualized strategies that address the psychological, social, economic, and cultural factors influencing eating patterns.

In agri-food systems, AI is already transforming how food is produced, processed, and managed. It helps predict crop yields, detect plant diseases at an early stage, and improve irrigation and fertilizer use. Across the food supply chain, AI can classify raw materials, monitor quality, estimate shelf life, optimize energy consumption, reduce waste, and strengthen traceability from farm to consumer (8). In nutrition settings, AI-driven diet recommendation systems are improving personalized nutrition by combining body composition data, cultural dietary patterns, and machine learning. One intelligent diet platform used parameters such as BMI, body fat percentage, and 3D body modeling to generate individualized meal plans, achieving a recommendation error rate below 3%. These findings demonstrate the potential of AI to provide more accurate, scalable, and culturally tailored dietary guidance compared with conventional one-size-fits-all approaches (9). These capabilities collectively suggest that AI could serve as a unifying force across the food and health continuum. AI-driven food and nutrition systems should be understood as a continuum rather than a set of isolated applications.

Artificial Intelligence (AI) is improving adaptive nutrition planning, self-management, and clinical decision-making by integrating personal health data into timely, individualized interventions. Technologies such as AI-enabled telemedicine and breathomics sensors support continuous monitoring of glucose, dietary intake, and respiratory biomarkers, thereby improving the management of chronic diseases such as diabetes, COPD, and CKD (10, 11). However, realizing the full potential of these technologies requires addressing critical challenges in data quality and standardization, algorithm interpretability, ethical governance, and health equity. Against this background, this perspective examines how AI is transforming the food–nutrition continuum, from nutritional intelligence and computational nutrition to smart food manufacturing, personalized nutrition, and multi-omics integration. Rather than providing another systematic review or meta-analysis, we present a forward-looking framework that synthesizes emerging advances, identifies key opportunities and implementation barriers, and highlights priorities for future research and clinical translation. Ultimately, we argue that the responsible integration of AI across the food–nutrition continuum can accelerate the development of more personalized, sustainable, resilient, and equitable food systems that benefit both human and planetary health.

## 2 The emerging architecture of AI-driven food and nutrition systems

Artificial Intelligence (AI) is increasingly conceptualized not as an isolated tool but as an integrated decision-making architecture that transforms diverse biological, nutritional, environmental, and industrial data streams into actionable insights across the food system (12). The rising burden of nutrition-related chronic diseases, including obesity, diabetes, and cardiovascular disorders, highlights the need to move beyond one-size-fits-all dietary recommendations toward personalized, data-driven nutritional approaches. Traditional dietary guidelines often overlook interindividual variations in genetics, metabolism, lifestyle, cultural practices, and environmental factors, limiting their effectiveness for many people. Concurrently, the food industry faces persistent challenges related to nutrient loss, limited supply-chain transparency, food safety risks, and inefficient resource utilization. Together, these challenges emphasize the need for smarter food systems that integrate advanced analytics, technological innovation, and personalized nutrition strategies to improve both food quality and human health (13).

At the core of AI-driven food systems is the integration of heterogeneous data sources, including imaging technologies, spectral sensing, process parameters, microbial and chemical safety indicators, and equipment telemetry. These interconnected data streams enable real-time food classification, quality monitoring, and process optimization. Deep learning approaches have demonstrated considerable potential in food recognition, grading, and safety assessment, with convolutional neural networks trained on large-scale food image datasets such as Food-101 representing early examples of AI-based food intelligence (14). Beyond individual applications, industrial AI platforms are increasingly enabling predictive quality control, early defect detection, and waste reduction by continuously learning from manufacturing and supply-chain data. This transition represents a shift from the traditional linear “farm-to-fork” model toward a dynamic, adaptive, and closed-loop food system. In this emerging architecture, data from food production, processing, distribution, consumption behavior, and health outcomes continuously feed back into AI models, enabling iterative improvement of decision-making in both food manufacturing and personalized nutrition. Such systems have the potential to simultaneously optimize food safety, nutritional quality, sustainability, and consumer health outcomes.

Beyond prediction, next-generation AI systems are evolving toward adaptive decision-support frameworks. These systems can learn from complex food environments and recommend optimized interventions. Technologies including artificial neural networks, federated learning, and genetic algorithms are increasingly being explored for applications in food processing, quality control, sorting, and process optimization. For example, AI-enabled self-optimizing Clean-In-Place (CIP) systems can improve hygiene management while reducing water and energy consumption. However, challenges such as climate change, shrinking agricultural land, and persistent micronutrient deficiencies continue to threaten global food security (15). Integrating AI with sustainability-oriented food strategies may enhance resource utilization, nutritional security, and resilience across the entire food supply chain.

The success of AI in food and nutrition systems is ultimately determined by the quality, representativeness, and reliability of the data that underpin its development. Models trained on incomplete food composition databases, inaccurate or biased annotations, underrepresented populations, or fragmented industrial datasets may achieve impressive computational performance while generating recommendations that are nutritionally misleading, culturally inappropriate, or impractical for real-world implementation (16). Therefore, robust data infrastructure, standardized data practices, and inclusive datasets are not merely technical improvements but fundamental prerequisites for ensuring scientific validity, translational reliability, and public trust in AI-driven food and nutrition systems.

## 3 Nutritional intelligence and personalized nutrition

**FIGURE 1:**
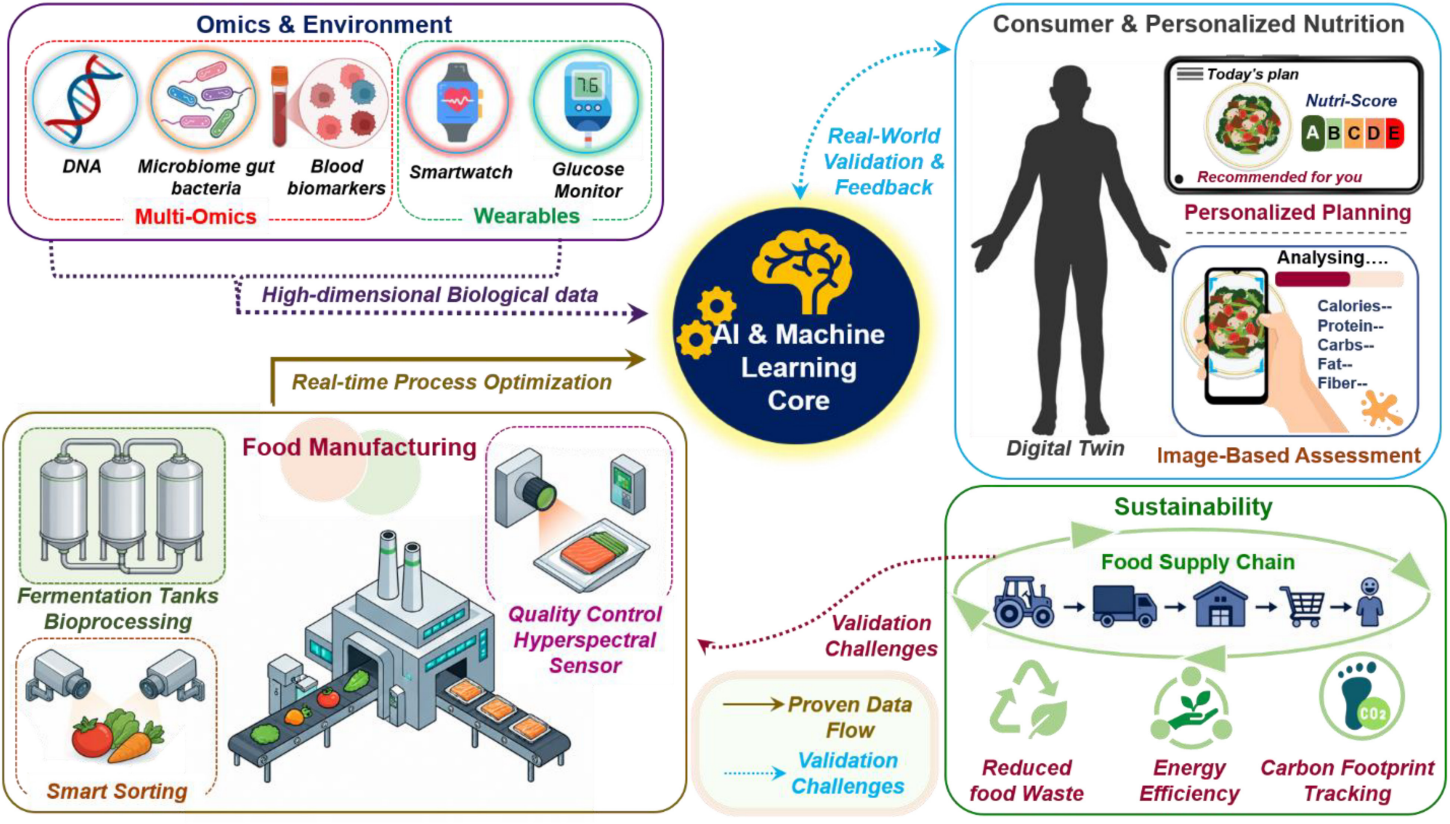
An AI-driven closed-loop framework for precision nutrition and sustainable food production. The central AI and Machine Learning Core (encompassing neural networks, LLMs, and computer vision) integrates multi-modal inputs from multi-omics and wearable biosensors (top left) to optimize food manufacturing via real-time process control, smart sorting, and fermentation bioprocessing (bottom left). Outputs feed into personalized consumer interfaces, including digital twin-enabled dietary planning and image-based meal assessment (right), with real-world feedback loops validating and refining core algorithms. A circular sustainability node (bottom right) tracks carbon footprint, waste reduction, and energy efficiency across the supply chain. Solid lines indicate established data flows; dashed lines denote validation challenges associated with biological and environmental variability.

Personalized nutrition is one of the most consumer-visible applications of AI, and a foundational use case is predicting individualized postprandial responses to dietary intake, as shown in Figure 1. Early evidence challenged the traditional assumption that individuals respond similarly to the same foods. In a landmark study involving 800 participants, Zeevi et al. monitored 46,898 real-world meals and continuous glucose responses over 1 week, revealing substantial interindividual variation in postprandial glycemic responses to identical foods. By integrating blood biomarkers, dietary patterns, anthropometric measurements, physical activity, and gut microbiome profiles, machine-learning models demonstrated the ability to predict individual glucose responses. These predictions were subsequently validated in an independent cohort of 100 participants, where an algorithm-guided personalized dietary intervention improved postprandial glucose control and influenced gut microbiome composition (17, 18). These findings provided early evidence that AI-driven nutrition strategies may overcome limitations of conventional population-based dietary recommendations by enabling individualized, data-driven dietary guidance.

Expanding this concept, the PREDICT 1 study by Berry et al. (19), involving 1,002 adults, demonstrated extensive interindividual variability in postprandial triglyceride, glucose, and insulin responses following standardized meals. Using machine-learning approaches incorporating personal, metabolic, and lifestyle characteristics, the study developed predictive models for individual dietary responses (19). Together, these studies established a key scientific foundation for personalized nutrition AI: individuals exhibit highly variable responses to the same foods, and a substantial proportion of this variability can be predicted using multimodal personal data. The successful translation of AI-driven personalized nutrition requires interdisciplinary collaboration among data scientists, nutrition researchers, clinicians, and healthcare professionals (20). While data scientists contribute computational frameworks for integrating complex biological and behavioral datasets, nutrition and clinical experts ensure that AI-generated recommendations remain scientifically valid, ethically responsible, and applicable in real-world settings.

### 3.1 AI methods for personalized dietary planning

AI-based dietary planning has evolved from conventional recommendation systems focused primarily on matching user preferences toward more intelligent models capable of integrating nutritional knowledge and individual health constraints. Yang et al. developed *Yum-me*, a personalized nutrient-based recommender system designed to balance nutritional requirements, dietary restrictions, and user food preferences. Building on this concept, Ma et al. introduced a nutrition-oriented knowledge graph neural network that integrates user preferences with recipe-level nutritional information to generate more context-aware recommendations (21). Together, these approaches demonstrate a transition from simple preference-based recommendation toward knowledge-driven systems capable of reasoning about food composition and nutritional suitability.

More recently, large language models (LLMs) have expanded the potential of AI-driven dietary planning by enabling interactive and context-aware nutrition guidance. Khamesian et al. (22) introduced NutriGen, an LLM-based framework that integrates personalized nutrition databases and external resources, including the USDA FoodData Central database, to generate dietary plans aligned with individual constraints (22). Similarly, Kopitar et al. (23) evaluated the ability of LLMs to decompose complex ingredients and support meal customization and nutritional assessment (23). These advances highlight the potential of LLMs to move personalized nutrition beyond predefined recommendation libraries. However, their reliance on accurate databases, expert validation, and proper clinical interpretation suggests that current systems should be used mainly as decision-support tools rather than as fully autonomous nutrition planners.

### 3.2 Image-based dietary assessment

Advances in computer vision, mobile computing, and artificial intelligence are transforming dietary assessment by enabling more automated, scalable, and objective approaches for monitoring food intake. AI-based dietary assessment systems primarily rely on text-based or image-based approaches, where textual descriptions or food images are analyzed to identify consumed foods and estimate their nutritional characteristics (24, 25). Early image-recognition systems demonstrated the feasibility of automated food identification, while recent deep learning approaches have substantially improved recognition accuracy and practical usability. Mezgec and Koroušić Seljak (26) developed NutriNet, which achieved high classification performance across hundreds of food and beverage categories, demonstrating the potential of AI for large-scale dietary monitoring (26). Similarly, Lu et al. (27) introduced goFOOD, a smartphone-based system integrating food detection, segmentation, recognition, and three-dimensional reconstruction to estimate portion size, energy, and macronutrient content from meal images (27). The system demonstrated performance comparable to or exceeding that of experienced dietitians across different meal datasets, highlighting the potential of AI-assisted dietary assessment for real-world applications.

Recent developments have further shifted the field from laboratory-based recognition toward deployable digital nutrition tools. Konstantakopoulos et al. (28) integrated convolutional neural networks (CNNs) with stereo vision approaches for Mediterranean food classification, volume estimation, and nutritional analysis (28). Meanwhile, hybrid CNN–vision transformer (ViT) architectures have improved fine-grained food recognition, with models such as DKTransformer and other CNN–ViT frameworks demonstrating strong performance across benchmark datasets and supporting mobile nutrition-analysis applications (29, 30). Collectively, these advances indicate that AI-based food recognition is transitioning from a computer vision challenge toward a practical digital nutrition infrastructure. However, challenges related to portion-size estimation, mixed dishes, cultural diversity of foods, image quality, and integration with personalized health data remain critical barriers for clinical and population-level implementation.

### 3.3 Real-world validation challenges

Despite rapid advances in AI-driven nutrition technologies, translating computational performance into reliable real-world applications remains a major challenge. Different AI approaches involve inherent trade-offs between interpretability, scalability, and predictive capability. Rule-based expert systems and fuzzy logic models offer greater transparency but depend on predefined assumptions and may fail in complex or unfamiliar scenarios. In contrast, artificial neural networks and convolutional neural networks can capture highly complex patterns from large datasets but require substantial computational resources, high-quality training data, and robust validation. Their limited interpretability remains a critical barrier for applications where explainability and accountability are essential (31).

Beyond algorithmic performance, AI systems must demonstrate measurable benefits in addressing practical challenges across the food system. Food waste represents a major global sustainability challenge; for example, the USDA Economic Research Service estimated that approximately 30–40% of the U.S. food supply is wasted, corresponding to nearly 133 billion pounds of food valued at USD 161 billion in 2010. AI-enabled approaches offer opportunities to reduce such losses through improved demand forecasting, inventory management, supply-chain optimization, early quality deterioration detection, and predictive decision-making. When integrated with sustainable agricultural and food production practices, AI can contribute to more efficient, resilient, and resource-conscious food systems (32). However, the effectiveness of these applications depends on reliable data streams, sensor integration, interoperability, and validation under diverse operational conditions.

Similarly, in personalized nutrition, high performance on computational benchmarks does not necessarily guarantee safe or clinically appropriate recommendations. Onay et al. (33) reported that ChatGPT-generated dietary plans for hypothetical chronic disease cases showed deficiencies in nutrient targets, limited personalization, and inclusion of potentially inappropriate foods (33). Likewise, Karakas et al. (34) evaluated six LLMs for type 2 diabetes meal planning and identified systematic nutrient biases, inconsistent guideline adherence, and substantial variability among models (34). These findings demonstrate that current AI systems should serve as decision-support tools rather than replacements for expert-guided nutritional care.

Consistent with this evidence, WHO’s 2021 guidance on AI for health stresses that AI systems must place ethics and human rights at the center of their design, deployment, and use, with clear accountability to health workers and affected communities (35). AI also supports smart packaging by detecting changes in food condition and possible interactions with nanomaterials. Cloud-based platforms allow producers, suppliers, and retailers to share data and improve traceability. Automated harvesting can reduce labor needs and increase productivity, while predictive tools can help balance supply and demand and strengthen hygiene monitoring.

Responsible deployment further requires addressing ethical governance, transparency, privacy, and equity. The WHO guidance on AI for health emphasizes that AI systems should prioritize human rights, accountability, safety, and fairness throughout their development and implementation (35). AI-driven personalized nutrition has great potential to improve dietary guidance, but its implementation requires clear accountability frameworks. AI recommendations should be validated, transparent, and supported by professional oversight. Developers and healthcare providers must ensure data privacy, reduce algorithmic bias, and prevent over-reliance on automated decisions. Responsible AI deployment in nutrition should balance innovation with safety, equity, and accountability (36). Additional barriers, including limited data sharing, poor interoperability, high implementation costs, and unequal access to digital infrastructure, may restrict adoption, particularly among smaller food businesses and underserved populations. Therefore, successful integration of AI into food and nutrition systems requires balancing predictive performance with interpretability, affordability, accessibility, and real-world applicability.

### 3.4 Omics, microbiome, and digital health integration

An emerging frontier in precision nutrition involves integrating dietary science with high-dimensional biological, behavioral, and environmental data through AI-driven analytical frameworks (Figure 1). Unlike conventional dietary approaches based primarily on food intake and anthropometric measurements, AI-enabled systems can incorporate genomics, metabolomics, proteomics, microbiome profiles, lifestyle information, and wearable sensor data to generate more dynamic models of individual nutritional responses (37, 38). This transition reflects a move toward digital phenotype-based nutrition, where continuously collected health information enables adaptive and personalized dietary recommendations.

Multi-omics integration has expanded our understanding of why individuals exhibit highly variable metabolic responses to identical foods. Findings from the PREDICT studies demonstrated substantial interindividual differences in postprandial glucose and lipid responses, influenced by a combination of microbiome composition, body composition, dietary patterns, and lifestyle factors (37). By integrating these complex datasets, AI provides new opportunities to identify response patterns and develop more precise nutrition strategies beyond traditional population-based dietary guidelines.

Computational nutrition has emerged as an interdisciplinary field that combines AI, systems biology, and nutritional science to predict individualized dietary responses, identify diet–health associations, model disease risk, and evaluate population-level nutrition strategies (39). However, translating these approaches into practical applications remains challenging due to limited population diversity, high-dimensional data complexity, insufficient causal understanding, and privacy concerns associated with genomic and behavioral datasets. Thus, implementing AI-enabled precision nutrition requires a structured validation framework that advances from computational modeling to controlled clinical studies and finally to real-world evaluation. It is essential to ensure transparency, external validation, and a clear separation between research applications and clinical recommendations.

## 4 AI in food manufacturing: efficiency, sustainability, and quality assurance

Food manufacturing represents one of the most mature application domains for AI (Figure 1), as modern production systems generate large volumes of image, sensor, process, and environmental data that can be leveraged for quality assurance, safety monitoring, and process optimization (40). Unlike conventional automation, the most established role of AI in food manufacturing is not simply replacing human operators but enabling data-driven decision-making to improve inspection accuracy, process stability, resource efficiency, and waste reduction. Agrawal et al. (41) highlighted that AI can enhance operational efficiency, strengthen quality control, minimize waste, and support circular-economy approaches within food manufacturing systems (41).

Recent advances indicate that AI applications are expanding from individual analytical tasks toward integrated digital manufacturing systems. Machine learning approaches are increasingly being applied in formulation optimization, process control, product quality prediction, predictive maintenance, intelligent packaging, shelf-life estimation, and cold-chain monitoring (42, 43). However, despite promising laboratory and pilot-scale results, the adoption of AI in commercial food manufacturing remains selective, with many applications still limited by challenges related to data availability, system integration, and validation under industrial conditions.

AI-based computer vision and sensing technologies represent some of the most developed applications in food quality and safety assessment. For example, Einsiedel et al. (44) evaluated object-detection models for ready-to-eat poultry products and reported high detection performance, with YOLO12 achieving a mAP50-95 score of 0.9359, while also highlighting the influence of class imbalance on model performance (44). Similarly, Aqeel et al. (45) combined hyperspectral imaging with machine-learning algorithms for milk adulteration detection and achieved complete validation accuracy within their multiclass dataset (45). At the system-integration level, Cui et al. demonstrated real-time seed classification by training deep learning models on 36,479 images and connecting analytical outputs with cloud platforms and manufacturing execution systems for adaptive optimization (46). Collectively, these studies demonstrate that AI-enabled inspection can achieve high performance in controlled manufacturing environments, but reliable deployment requires robust labeling strategies, representative datasets, and domain-specific validation.

Beyond inspection, AI is increasingly shifting toward proactive process optimization by predicting and controlling production dynamics before quality losses occur. In brewery operations, Liu et al. (47) demonstrated that AI-based steam-flow control reduced process overshoot, improved filtration efficiency, increased recovery yield, and shortened processing time across multiple production batches (47). Similarly, Fernández-Peláez et al. (48) applied vibration monitoring and machine learning to predict equipment failures in food-processing machinery, supporting maintenance decisions under real operational conditions (48). These examples highlight that the greatest industrial value of AI may lie not only in identifying defects but also in preventing inefficiencies through predictive and adaptive control.

The sustainability potential of AI is most convincing when linked to measurable improvements in energy use, emissions, and resource efficiency. Redchuk et al. (49) developed AI-based optimization models for steam generation in a food ingredient manufacturing facility using operational and environmental data. This model identified key drivers of fuel consumption and reduced fuel costs by 4% while lowering greenhouse-gas emissions by more than 10 million pounds of CO2 annually (49). Such applications demonstrate that AI can contribute to sustainable food manufacturing when integrated with clearly defined engineering objectives and evaluated using measurable environmental outcomes.

Despite these advances, large-scale implementation remains challenging. Barriers include integration with legacy manufacturing systems, limited availability of skilled personnel, data privacy concerns, algorithmic bias, and insufficient model transparency. In high-stakes food production environments, explainability and reliability are essential for regulatory compliance, safety assurance, and operator trust. Therefore, the future progress of AI in food manufacturing will depend not only on improving model performance but also on developing interoperable, explainable, and deployment-ready systems capable of operating reliably in diverse industrial environments.

## 5 Conclusion

Artificial Intelligence (AI) is emerging as a transformative force across the food and nutrition system, with the potential to accelerate food innovation, enhance production efficiency, strengthen quality assurance, reduce food loss and waste, support sustainability, and enable more personalized dietary guidance. Across the agri-food value chain, AI improves decision-making, increases supply-chain transparency, and supports the development of precision nutrition approaches. However, its most important role is not simply predictive but integrative. Modern food systems generate diverse and fragmented datasets spanning agriculture, processing, retail, dietary behaviors, and health outcomes. AI provides an opportunity to connect these domains within unified decision-making frameworks, but realizing this potential will require robust data governance, interdisciplinary collaboration, and rigorous validation to ensure scientific and nutritional integrity.

A central challenge is balancing computational capability with nutritional trustworthiness. Although applications such as AI-based meal planning, food image recognition, process optimization, and formulation design are becoming technically feasible, their real-world impact depends on safety, interpretability, contextual relevance, and demonstrated health benefits. The availability of AI tools alone does not guarantee nutritional validity or clinical applicability. Similarly, balancing scalability with personalization remains a critical consideration, as AI systems excel at large-scale data analysis, whereas nutrition science requires recognition of individual biological, cultural, and environmental variability. Addressing this challenge will require diverse and representative datasets, domain-informed modeling, expert oversight, and meaningful collaboration among clinicians, dietitians, food scientists, industry stakeholders, and consumers.

The future of AI-driven food systems lies in the development of closed-loop frameworks that connect food production, manufacturing, consumption patterns, and health outcomes. These systems have the potential to improve adaptability, sustainability, and resilience across the food chain while enabling more personalized approaches to nutrition and health. However, their responsible implementation will require continued attention to transparency, privacy, accountability, cost, and equitable access. Ultimately, AI should evolve as a human-centered enabling technology that enhances rather than replaces scientific and professional expertise, enabling safer, more sustainable, equitable, and evidence-based food systems for improved human and planetary health.

## Data Availability

The original contributions presented in this study are included in the article/supplementary material, further inquiries can be directed to the corresponding author.
